# Use of the LI-RADS classification in patients with cirrhosis due to infection with hepatitis B, C, or D, or infected with hepatitis B and D

**DOI:** 10.1590/0100-3984.2018.0077

**Published:** 2020

**Authors:** Rita de Cassia Ribeiro Pereira, Carolina Augusta Modena Heming, Thiago Ramos Tejo, Thais Cristina Lima de Oliveira, Rita do Socorro Uchoa da Silva, Daniella Braz Parente

**Affiliations:** 1 Universidade Federal do Acre (UFAC), Rio Branco, AC, Brazil.; 2 Instituto Nacional de Câncer José Alencar Gomes da Silva (INCA), Rio de Janeiro, RJ, Brazil.; 3 Universidade Federal do Rio de Janeiro (UFRJ), Rio de Janeiro, RJ, Brazil.; 4 Instituto D’Or de Pesquisa e Ensino (IDOR), Rio de Janeiro, RJ, Brazil.

**Keywords:** Carcinoma, hepatocellular, Liver cirrhosis, Tomography, X-ray computed

## Abstract

**Objective:**

To evaluate liver lesions, in accordance with the LI-RADS classification, using contrast-enhanced multiphase dynamic computed tomography in patients with hepatitis B, coinfected or not with hepatitis D, or with chronic hepatitis C, as well as to determine the level of agreement between radiologists.

**Materials and Methods:**

We evaluated 38 patients with hepatitis B, coinfected or not with hepatitis D, or with chronic hepatitis C, all of whom underwent contrast-enhanced multiphase dynamic computed tomography. For each examination, two radiologists selected up to three hepatic lesions, categorizing them in accordance with the LI-RADS classification and evaluating signs of chronic liver disease and portal hypertension. To determine the level of agreement between radiologists, we calculated the kappa statistic (κ) .

**Results:**

Radiologist 1 and radiologist 2 selected 56 and 48 liver lesions, respectively. According to radiologist 1 and radiologist 2, respectively, 27 (71%) and 23 (61%) of the 38 patients had at least one liver lesion; 13 (34%) and 12 (32%) had a LI-RADS 5 lesion (κ = 0.821); 19 (50%) and 16 (42%) had a hypervascular lesion (κ = 0.668); and 30 (79%) and 24 (63%) had splenomegaly (κ = 0.503). Both radiologists identified chronic liver disease in 31 (82%) of the patients (κ = 1.00).

**Conclusion:**

Lesions categorized as LI-RADS 5 were detected in approximately 32% of the patients, with almost perfect agreement between the radiologists. The level of agreement was substantial or moderate for the other LI-RADS categories.

## INTRODUCTION

In Brazil, the estimated incidence of hepatocellular carcinoma (HCC) in 2016 was 10,000 cases, with a crude mortality rate of 5.1/100,000 population, making it the sixth leading cause of cancer-related death in the country^(1,2)^. The main risk factors for HCC are infection with the hepatitis B virus (HBV) and liver cirrhosis, which is present in up to 90% of patients with HCC^(3,4)^. The main causes of hepatic cirrhosis are chronic viral hepatitis caused by infection with the hepatitis B virus or the hepatitis C virus (HBV and HCV, respectively), alcoholic cirrhosis, and nonalcoholic fatty liver disease^(4)^. It is known that HBV is carcinogenic and can lead to the development of HCC even in the absence of cirrhosis^(5)^.

For individuals in the population groups at risk of developing HCC, it is common to undergo abdominal ultrasound screening every six months either with or without determination of the alpha-fetoprotein level. When a focal lesion ≥ 1 cm is identified in the abdominal ultrasound screening, the use of a cross-sectional imaging method, such as contrast-enhanced multiphase dynamic computed tomography (CT) or magnetic resonance imaging (MRI), is indicated in order to confirm the diagnosis and staging^(6)^.

In 2008, the American College of Radiology created a system of data and reports known as the Liver Imaging Reporting and Data System (LI-RADS), which has high specificity for the diagnosis of HCC^(7-12)^, in order to standardize the descriptions of hepatic lesions in cirrhosis among radiologists and to facilitate communication within multidisciplinary groups.

In the present study, we evaluated the hepatic lesions identified by contrast-enhanced multiphase dynamic CT in accordance with the LI-RADS classification in patients with chronic HCV or with HBV, coinfected or not with the hepatitis delta virus (HDV), and looked for signs of chronic liver disease. We also gauged the level of interobserver agreement.

## MATERIALS AND METHODS

This was an analytical, cross-sectional, observational study, involving a convenience sample of 38 patients with chronic hepatitis C or with hepatitis B, coinfected or not with HDV, treated as inpatients or outpatients at Hospital das Clínicas de Rio Branco, in the state of Acre (in northwest Brazil), between April and December of 2017. All of the patients underwent contrast-enhanced multiphase dynamic CT. Epidemiological data were collected by interview on the day of the examination or from medical records. Pregnant women were excluded from the study, as were patients younger than 18 years of age and patients with contraindication to the contrast medium. The study was approved by the local research ethics committee (Reference no. 58423116.2.0000.5010).

The CT scans were acquired in a 16-slice multidetector scanner (Brivo; GE Healthcare, Chicago, IL, USA) and the protocol followed was that for the upper abdomen with helical acquisitions before and after administration of intravenous contrast medium with a dynamic study, in accordance with the American College of Radiology recommendations for application of the LI-RADS 2018 version^(6)^, with a slice thickness of 1.5 mm. We used nonionic iodinated contrast (Omnipaque 300 mgI/mL; GE Healthcare, Shanghai, China) at a dose of 1.2 mL/kg, administered by injection pump at a rate of 3.0 mL/s, and a control tool to trigger the contrast for acquiring the contrast-enhanced images in the arterial, portal, and equilibrium phases. The acquisition control of the arterial phase was based on a density of 180 HU measured in the region of interest located in the transition from the thoracic aorta to the abdominal aorta. The portal phase and equilibrium phase images were acquired at 40 s and 180 s, respectively, after the end of the arterial phase.

The imaging findings of the liver were analyzed by two radiologists (radiologist 1 and radiologist 2, each with seven years of experience), working independently, who classified the hepatic lesions, referred to as hepatic observations in the present study, according to the criteria established in the LI-RADS version 2018. Signs of portal hypertension, hepatomegaly, collateral circulation, ascites, splenomegaly, and chronic liver disease were also evaluated.

**Figure 1:**
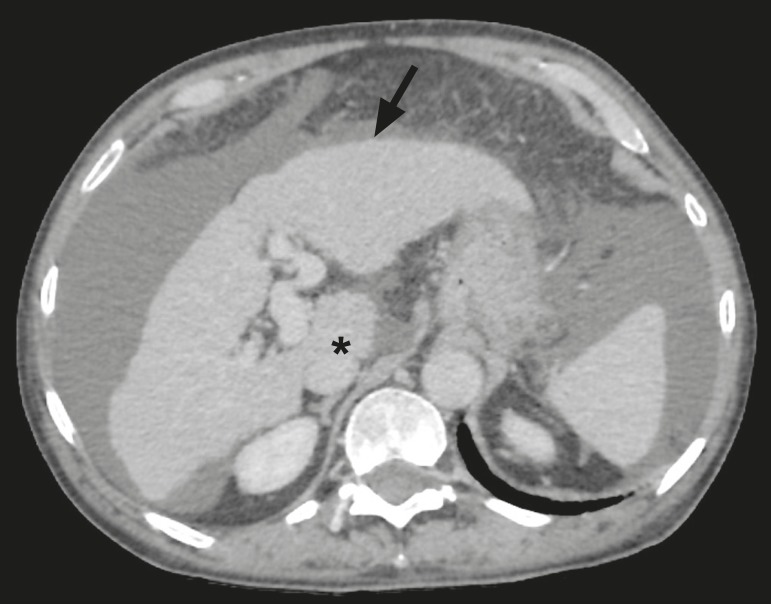
Findings indicative of chronic liver disease. CT in the portal phase, with signs of chronic liver disease: widened fissures, heterogeneity of the hepatic parenchyma, irregular contours, with prominence of lateral segments of the left lobe (arrow) and of the caudate lobe (asterisk), and atrophy of segment IV and of the right lobe. Note also the signs of portal hypertension, including ascites and increased portal vein diameter.

The qualitative criteria used in considering signs of chronic liver disease were widened fissures, heterogeneity of the parenchyma, and irregular contours (Figure 1). Advanced cases of hepatic cirrhosis were identified based on hypertrophy of the caudate lobe, as well as of segments II and III, with atrophy of segment IV and of the right lobe^(13)^.

Hepatomegaly was defined as the left lobe of the liver measuring > 6 cm along its longest (anteroposterior) axis and the right lobe measuring > 16 cm along its longest (longitudinal) axis^(13)^. Splenomegaly was identified by determining the splenic index (multiplying the major axes of the spleen: longitudinal × anteroposterior × transverse), which has an upper limit of normality of 480^(14)^.

**Figure 2:**
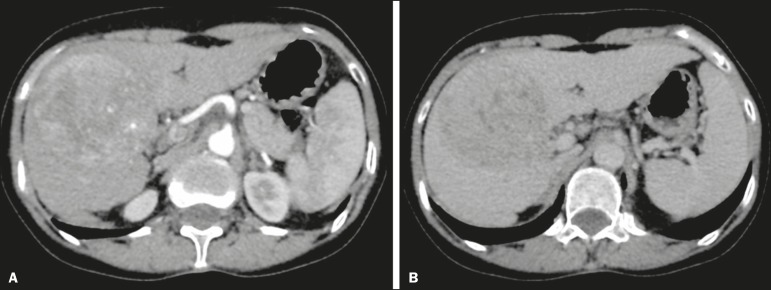
LI-RADS 5 observation. CT in the arterial and portal phases (**A** and **B**, respectively) showing a hypervascular observation > 20 mm (**A**), with washout (**B**), in a patient with hepatitis B.

Up to three hepatic observations were selected from each examination (observation 1, observation 2, and observation 3), categorized in descending order from the largest in the highest LI-RADS category to the smallest in the lowest. The lesions were evaluated for the presence of the major HCC criteria: hypervascularity, washout, and pseudocapsule (Figure 2). Each radiologist was blinded to the interpretation of the other. 

The data collected were analyzed with the IBM SPSS Statistics software package, version 20.0 (IBM Corp., Armonk, NY, USA). The kappa statistic (κ) was calculated to assess the level of agreement between the imaging findings and the LI-RADS categories. The level of agreement was categorized, in accordance with the kappa statistic, as follows^(15)^: perfect (κ = 1); almost perfect (κ, 0.81-0.99); substantial (κ, 0.61-0.80); moderate (κ, 0.41-0.60); fair (κ, 0.21-0.40); and slight (κ, 0.01-0.20).

To evaluate the level of agreement for the observation 1 measurements, splenic index, measurements of the right hepatic lobe, and measurements of the left hepatic lobe, we used Pearson’s correlation coefficient (r). For the value of r (strength of the linear correlation), we considered the following categories^(16)^: 0.00 < r < 0.30 (weak); 0.30 ≤ r < 0.60 (moderate); 0.60 ≤ r < 0.90 (strong); and 0.90 ≤ r < 1.00 (very strong).

For the clinical evaluation of patients, we employed the Child-Pugh scoring system^(17)^, which classifies the severity of chronic liver disease on the basis of biochemical values (albumin, bilirubin, and prothrombin time) and clinical findings (presence of ascites and hepatic encephalopathy), the score ranging from 1 to 3 for each item. For each patient, the disease was categorized as Child-Pugh class A (5-6 points), B (7-9 points), or C (10-15 points), a lower score indicating a better prognosis.

## RESULTS

The study included 38 patients, 25 (66%) of whom were male. Ages ranged from 19 to 75 years (mean, 50 ± 14 years). All 38 patients had viral hepatitis: 12 (32%) had HBV only; 11 (29%) were coinfected with HBV and HDV; 11 (29%) had chronic HCV only; one (3%) was coinfected with HBV and HCV; and one (3%) was coinfected with HBV, HCV, and HDV. Overall, 25 (66%) of the patients had HBV and 13 (34%) had chronic HCV.

It was possible to calculate the Child-Pugh score in 14 patients, among whom the disease was categorized as Child-Pugh class A in 11 (79%) and as Child-Pugh class B in 3 (21%). For the remaining patients, the examinations did not yield enough information to calculate the Child-Pugh score.

The most frequent common findings were signs of chronic liver disease (in 82%; κ = 1.00) and splenomegaly (in 63% and 79%; κ = 0.503). Therefore, there was perfect agreement in identifying the signs of chronic liver disease (Table 1).

**Table 1:** CT imaging findings.

	Radiologist 1			Radiologist 2		Agreement
Imaging finding	N	%		N	%	(κ)
Chronic liver disease	31	82%		31	82%	1.000*
Splenomegaly	30	79%		24	63%	0.503^§^
Collateral circulation	24	63%		25	66%	0.713^‡^
Hepatomegaly	11	29%		12	32%	0.564^§^
Ascites	10	26%		8	21%	0.855^†^
No. of hepatic lesions						0.421^§^
0	11	29%		15	40%	
1	9	24%		9	24%	
2-3	8	21%		7	18%	
4-6	4	11%		2	5%	
≥ 7	6	16%		5	13%	

* Perfect agreement;

† Almost perfect agreement;

‡ Substantial agreement;

§ Moderate agreement.

We selected 56 observations made by radiologist 1 and 48 observations made by radiologist 2. At least one liver lesion was detected (observation was made) by radiologist 1 and radiologist 2 in 71% and 60% of the patients, respectively. There was substantial agreement on the location of the majority of the lesions (κ = 0.723), most of which were found in the right lobe. Of the patients in whom there were no focal hepatic observations, 71% were categorized as having Child-Pugh class A disease.

Radiologist 1 identified 27 hepatic lesions and radiologist 2 identified 23, all considered observation 1 lesions, with substantial agreement regarding the LI-RADS classification (κ = 0,615; Table 2). The measurements of the observation 1 lesions had a strong correlation (*p* = 0.721). Radiologist 1 categorized 13 (48%) of the 27 observation 1 lesions as LI-RADS 5, whereas radiologist 2 categorized 12 (52%) of the 23 observation 1 lesions as LI-RADS 5. Therefore, the interobserver agreement for LI-RADS 5 lesions was almost perfect (κ = 0.821).

**Table 2:** Frequency of LI-RADS in the observation 1 lesions in abdominal CTs of patients with liver cirrhosis or HBV.

	Radiologist 1			Radiologist 2		Agreement
Imaging findings	N	%		N	%	(κ)
LR observation 1						0.615*
LI-RADS 1	1	4%		2	9%	
LI-RADS 3	9	33%		3	13%	
LI-RADS 4	2	7%		4	17%	
LI-RADS 5	13	48%		12	52%	
LI-RADS TIV	2	7%		0	0%	
LI-RADS M	0	0%		2	9%	

LR observation 1, LI-RADS classification of observation 1 lesions.

* Substantial agreement.

There was disagreement regarding the LI-RADS category in 11 (29%) of the 38 examinations. Of those 11 lesions, 6 (55%) and 3 (27%) were < 2 cm and < 1 cm, respectively. In addition, 4 (36%) of those 11 lesions were categorized as LI-RADS 3 by radiologist 1 and were not identified by radiologist 2. All four of those lesions were hypervascular, probably representing perfusion disorders. Three patients presented observation 2 lesions meeting the LI-RADS 5 criteria, with substantial agreement between the two radiologists in relation to the classification of the categories of those observations (κ = 0.658), as can be seen in Table 3).

**Table 3:** Frequency of LI-RADS in observation 2 and 3 lesions in abdominal CTs of patients with liver cirrhosis or HBV.

	Radiologist 1			Radiologist 2		Agreement
Imaging findings	N	%		N	%	(κ)
LR observation 2						0.658
LI-RADS NC	1	2.6%		0	0.0%	
LI-RADS 1	0	0.0%		1	2.6%	
LI-RADS 3	9	23.7%		6	15.8%	
LI-RADS 4	4	10.5%		2	5.3%	
LI-RADS 5	3	7.9%		3	7.9%	
LI-RADS TIV	0	0.0%		1	2.6%	
LI-RADS M	1	2.6%		1	2.6%	
LR observation 3						0.433
LI-RADS 1	0	0.0%		1	2.6%	
LI-RADS 2	0	0.0%		1	2.6%	
LI-RADS 3	11	28.9%		9	23.7%	
LI-RADS 4	2	5.3%		0	0.0%	

LR observation 2, LI-RADS classification of observation 2 lesions; LR observation 3, LI-RADS classification of observation 3 lesions.

In relation to the major criteria identified in observation 1 lesions, the two radiologists collectively identified hypervascular lesions in 70% of the observations selected and evaluated (κ = 0,668; Table 4), such lesions being identified be radiologists 1 and 2 in 50% and 42% of the patients, respectively. In the analysis performed by radiologist 1, 42% of hypervascular observations and 50% of the observations considered as washout were in patients with HCV, while all pseudocapsule observations were in patients with HBV only. Regarding the evaluation by radiologist 2, we observed that 44% of the hypervascular observations and 54% of the observations with washout were in patients with HCV, whereas 67% of pseudocapsule observations were in patients coinfected with HBV and HDV.

**Table 4:** Frequency of major LI-RADS criteria in observation 1 lesions.

	Radiologist 1			Radiologist 2		Agreement
Major criteria	N	%		N	%	(κ)
Hypervascular	19	70%		16	70%	0.668*
Washout	14	52%		13	57%	0.569^†^
Pseudocapsule	3	11%		3	13%	0.574^†^

* Substantial agreement;

† Moderate agreement.

## DISCUSSION

Recent studies in the radiology literature of Brazil have emphasized the importance of imaging examinations in the evaluation of hepatic neoplasms^(18-23)^. The present study used the LI-RADS classification system to analyze 38 patients infected with hepatitis viruses (HBV, HCV, HDV, or combinations thereof) who underwent CT. We found that approximately 32% of the patients presented with LI-RADS 5 observations (i.e., lesions diagnosed by imaging as classic HCC). We also observed substantial to moderate agreement between the two evaluators in terms of the LI-RADS categories. These findings demonstrate not only how important it is for reports to be systematized for diagnosing HCC but also how important it is to have good communication within a multidisciplinary team.

Because the LI-RADS classification system is recent, there have been only a few studies using the LI-RADS with verification of the interobserver agreement. Unlike our findings, those obtained by Barth et al.^(15)^ showed slight to moderate interobserver agreement for categorizing hepatic lesions by means of the LI-RADS. That discrepancy can be explained by the superior training and practice with the LI-RADS classification on the part of the radiologists, as well as by the smaller number of radiologists involved, in the present study. The selection of higher categories (LI-RADS 4 and LI-RADS 5) could also explain the higher levels of agreement observed in our study. Our findings are similar to those of Zhang et al.^(24)^, who also used a smaller number of radiologists and found no statistically significant difference between two groups of readers in terms of the LI-RADS categories. Greater experience with the LI-RADS classification on the part of the radiologists is important for greater interobserver agreement.

The LI-RADS classification should be used on a routine basis by radiologists, with a learning curve and accumulation of experience^(15)^, as has occurred with other well-established classification systems, such as the Breast Imaging Reporting and Data System^(15,25)^. It has been shown that training and experience in applying the Breast Imaging Reporting and Data System improved the level of agreement and the performance of radiologists^(26)^. Studies have indicated the need to refine and reduce the complexity of the LI-RADS classification, which will only be possible if it is more widely disseminated. It is expected that the routine use of this classification system will improve the level of agreement and the performance of radiologists. 

**Figure 3:**
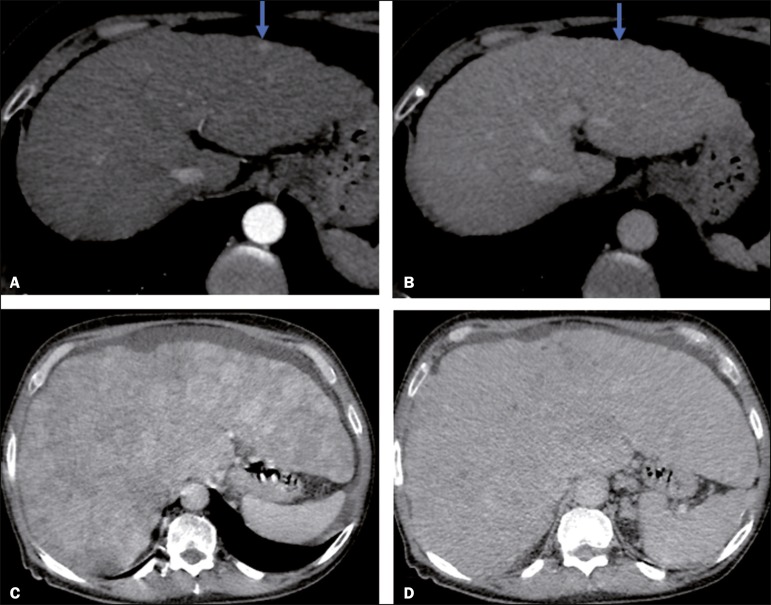
Cases of lower agreement between the radiologists. **A,B**: Images acquired in the arterial (**A**) and portal (**B**) phases, in a patient with a hypervascular lesion < 1 cm (arrow), categorized as LI-RADS 3. The smaller dimensions and difficulty in making a precise evaluation of the presence or absence of washout impede detection and classification. **C,D**: Images acquired in the arterial (**C**) and portal (**D**) phases, in a patient with multiple observations, mostly hypervascular, with partially defined and confluent borders, which hinder the selection and measurement of the observations, even the largest (> 2.0 cm) and most well-defined ones, and the identification of washout in these observations; the larger lesions were categorized as LI-RADS 4.

In the present study, using the major criteria of the LI-RADS, we found almost perfect agreement in the identification of LI-RADS 5 lesions, which demonstrates the ease in the identification of classic HCC by radiologists, a lesion that is treatable without biopsy, as advocated by the American College of Radiology. We observed greater disagreement in the classification of lesions < 2 cm and LI-RADS 3 observations-the intermediate risk category. When we evaluated the agreement only for the observation 3 lesions (those with smaller dimensions and in a lower LI-RADS category), among which the LI-RADS 3 category was more common, the level of agreement fell from substantial to moderate. Two other studies also reported greater difficulty in characterizing LI-RADS 3 lesions^(15,24)^. In one of those studies, using MRI, the association between the dimensions of the lesion and the level of interobserver agreement for the LI-RADS categories was evaluated and the authors found no difference in the assessment of major and minor lesions^(15)^. The use of MRI, which is suited to tissue characterization, can explain the difference in results. The LI-RADS 3 category presents intermediate probability of HCC. The observations that are too mild to meet the criteria for LI-RADS 2 (probably benign) and too severe to meet the criteria for LI-RADS 4 (probably HCC) are classified as LI-RADS 3. Therefore, when the diagnosis is questionable, there is also a tendency towards choosing the category with a lower degree of certainty (i.e., LI-RADS 3). Therefore LI-RADS 3 lesions will always be the ones for which there is the most diagnostic uncertainty (Figure 3).

The present study reports that interobserver agreement was substantial for the identification of hypervascular observations and moderate for the identification of washout and a pseudocapsule (major criteria). We also found a strong correlation between the dimensions of the lesions and the level of interobserver agreement. Our findings are in agreement with those of Barth et al.^(15)^ and Bashir et al.^(27)^, both of whom reported moderate interobserver agreement for the major LI-RADS criteria and for the classification of observations, as well as reporting excellent agreement for the measurements of the lesions when comparing hypervascular lesions on CT and MRI in patients at risk for HCC. Although we identified a strong correlation between the dimensions of the lesions and the level of interobserver agreement, it was weaker than that described by Barth et al.^(15)^ and Bashir et al.^(27)^-both of whom used information from MRI-a method that is more accurate and more suited to tissue characterization, which facilitates the delimitation of the lesions and may explain the difference in correlation when compared to our work. Despite CT being less suited to tissue characterization than is MRI, it is also effective in the diagnosis of HCC, including the infiltrative type, a lesion that is more difficult to delimit, as was also observed among our patients with HBV/HDV coinfection, in the state of Acre, and may have hindered the measurement of the lesions by the radiologists in our study.

In the present study, hepatic cirrhosis was the most common imaging finding identified by radiologists, who were in perfect agreement for this aspect. It is known that hepatic cirrhosis is an important risk factor for HCC^(3,4)^. It is worth mentioning that the radiological signs of chronic liver disease are not clearly revealed in its initial phase, at which time the liver may still have a normal morphological aspect, but are evident in its later stages^(28)^, which could explain the high level of interobserver agreement for its diagnosis.

Splenomegaly, which is common in patients with portal hypertension^(29-31)^, was the second most common imaging finding in the present study, and the level of agreement was only moderate, even with the use of the splenic index. Measurements along the longer axes of the spleen in different planes could explain the different interpretations. It should be emphasized that splenomegaly is one of the findings that characterize portal hypertension, and it has been associated with a worse prognosis^(29)^.

One peculiarity of our study in relation to others that used the LI-RADS classification was the presence of patients infected with HDV (44% of those infected with HBV, corresponding to 29% of all of the patients evaluated). We believe that the high rate of HBV/HDV coinfection was related to the study population, because the rate of HBV/HDV coinfection is high in Acre. The patient selection process might also have played a role, because the study was conducted at a referral center for infectious diseases, which in itself could lead to overestimation of the coinfection rate. Studies have shown a high prevalence of the HDV in the population of the western Brazilian Amazon, which includes the state of Acre (where there is a high frequency of the viral forms of hepatitis, especially in the interior of the state) and the state of Amazonas, the two states in which the patients in our sample resided^(32-34)^. It is believed that the high frequency of hepatitis B reflect failures in vaccination campaigns in that region^(32)^. The hepatocarcinogenic effect of these hepatitis viruses is well known^(35,36)^, including the increased risk of HCC in cases of coinfection^(36,37)^. In our study, the frequency of LI-RADS 5 lesions was greater in the patients with hepatitis B, although that might have just been due to the larger number of individuals infected with the virus in the sample.

One of the limitations of our study is that we used CT rather than MRI for the evaluation of hepatic lesions. We chose to use CT because MRI is an expensive imaging method and not widely available in the northern region of Brazil. A limitation inherent to the use of CT is its inability to evaluate the ancillary features addressed in the 2018 version of the LI-RADS. In addition, the small sample size precluded the analysis of a possible correlation between LI-RADS 5 lesions and HBV/HDV coinfection.

In conclusion, the level of interobserver agreement between our two radiologists was substantial to moderate for the classification of the observations into LI-RADS categories and almost perfect for the presence or absence of a LI-RADS 5 lesion. Our findings suggest that the LI-RADS classification can be an important tool for the diagnosis of classic HCC, which is treatable without biopsy, and can improve the analysis of and diagnosis based on these imaging findings by standardizing radiology reports and improving understanding by the multidisciplinary team.
